# miRNA-Mediated Low Expression of EPHX3 Is Associated with Poor Prognosis and Tumor Immune Infiltration in Head and Neck Squamous Cell Carcinomas

**DOI:** 10.1155/2022/7633720

**Published:** 2022-04-01

**Authors:** Shun Ding, Qichao Hong, Tingting Duan, Zhengyang Xu, Qing He, Dongqin Qiu, Lin Li, Jingren Yan, Qimeng Zhang, Zhonglin Mu

**Affiliations:** ^1^Department of Otolaryngology, Head and Neck Surgery, The First Affiliated Hospital, Hainan Medical University, Haikou 570102, China; ^2^Department of Otorhinolaryngology Head and Neck Surgery, Hainan General Hospital (Hainan Affiliated Hospital of Hainan Medical University), Haikou 570311, China

## Abstract

The aim of this study was to explore the regulatory role of epoxide hydrolase 3 (EPHX3) in head and neck squamous cell carcinoma (HNSCC) and to analyze its bioinformatic function, as well as, to screen and predict the miRNAs that can regulate EPHX3 expression in HNSCC. We examined the expression profile and prognostic potential of EPHX3 in TCGA and GTEX databases and performed functional enrichment analysis of EPHX3 using string database. Subsequently, we analyzed the regulatory role of miRNAs on EPHX3, including expression analysis, correlation analysis, and survival analysis. In addition, we also used TIMER to investigate the relationship among EPHX3 expression level, immune checkpoints, and immune infiltration in HNSCC. The results of data analysis after TGCA showed that EPHX3 is a key regulator of tumorigenesis in 13 cancers and can be used as a marker of poor prognosis in HNSCC patients. Bioinformatics analysis revealed that miR-4713-3p is a key miRNA of EPHX3 in HNSCC. Together, our findings indicate that EPHX3 exerts its anticancer effects by suppressing tumor immune checkpoint expression and immune cell infiltration. Overall, our data uncovered miRNA-mediated EPHX3 downregulation as a contributor to poor HNSCC prognosis and reduced tumor immune infiltration.

## 1. Introduction

Over the past 2020, the number of newly reported head and neck cancer cases worldwide has been approximately 740000 [1]. Head and neck squamous cell carcinoma (HNSCC) is the commonest cancer type affecting the head and neck region and may originate from the mucosal epithelium of the oral cavity, pharynx, or larynx [2]. It is the 7th most prevalent cancer worldwide at a mortality rate of 40-50% [3]. HNSCC is mainly treated using surgery, radiation, chemotherapy, or a combination of these. However, these treatments are often unsuccessful and significantly lower the patient's quality of life. In particular, salvage surgery is lengthy and mutilating for the patient [4]. Thus, effective HNSCC therapeutic targets or prognostic biomarkers are urgently needed.

Epoxide hydrolases are a small family of *α*/*β* hydrolase fold enzymes that break down chemically reactive xenobiotic epoxides and process endogenous epoxides that act as signaling molecules. EPHX3 is an epoxide hydrolase that contains a highly conserved 16-amino acid motif and is abundantly expressed in the proximal digestive system, bone marrow, lymphoid tissues, and skin [5]. EPHX3 expression has been associated with increased risk of some malignancies, such as gastric cancer [6], melanoma [7], and prostate cancer [8]. Hypermethylation in the promoter region was linked to a lower transcription rate. Recent findings that epoxyeicosatrienoic acids (EETs) can reawaken latent tumors and promote metastasis, presumably through their proangiogenic characteristics [9, 10], imply that EPHX3 might have anticancer functions. However, there are no studies on the role of EPHX3 in HNSCC.

Here, we investigated EPHX3 expression levels and association with survival in various human cancers. We then subjected EPHX3 to functional enrichment analysis, followed by assessment of its regulatory miRNAs in HNSCC. Finally, we investigated the association between EPHX3 expression and immune infiltration, immune cell biomarkers, and immune checkpoints in HNSCC. Our data show that miRNA-mediated EPHX3 suppression correlates with poor prognosis and immune cell infiltration in HNSCC patients.

## 2. Materials and Methods

### 2.1. Data Sources and Analyses

About 33 human cancer type information data are contained within The Cancer Genome Atlas (TCGA) database in the Genomic Data Sharing (GDC) data portal [11]. We downloaded these tumor RNA SEQ data and matched normal tissue samples.

Using the R package (beanplot) to analyze the different expression of EPHX3 in pan-cancer, statistical analyses were done on R 4.0.3. Unless otherwise specified, only two groups of data were identified by rank sum test. *p* < 0.05 indicated statistically significant differences.

### 2.2. Protein-Protein Interaction (PPI) Network Functional Enrichment Analysis

The interactions between proteins constitute a major component of cellular biochemical reaction networks. The STRING database (https://string-db.org/) is a database that searches online for interaction relationships between known proteins [12]. The 50 functional genes most closely related to EPHX3 were searched in STRING's “Multiple Proteins” module, requiring a minimum interaction score of 0.4 and imported into Cytoscape to construct a PPI network. Finally, we used the STRING database for functional enrichment analysis of genes associated with EPHX3 with *p* < 0.05 indicating statistical significance.

### 2.3. Analysis of the GEPIA Database

GEPIA (http://gepia.cancer-pku.cn/) is a newly developed web server for tumor/normal differential expression analysis and interactive analysis [13]. Analysis of the correlation between EPHX3 expression and survival in different cancer types was done using GEPIA, combining overall survival (OS) and disease-free survival (RFS). In addition, we also evaluated the correlation of EPHX3 expression with immune checkpoints in HNSCC utilizing the GEPIA database.

### 2.4. Prediction of miRNA Candidates

miRNAs are widely involved in the negative regulation of target genes. Here, we used TargetScan, TarBase, MIRDB, and miRmap to identify miRNAs that might modulate EPHX3 function and retained the ones that were in common between the different tools for subsequent analysis. TargetScan (http://www.targetscan.org/) looks for conserved 8mer, 7mer, and 6mer sites that match the seed region of each miRNA to predict biological targets for miRNAs [14]. TarBase (http://www.miRNA.gr/tarbase) is a database dedicated to indexing experimentally validated miRNA targets [15]. MiRDB (http://www.mirdb.org/) is a web-based database that predicts miRNA targets and provides functional annotations. MirTarget, a bioinformatics tool that was built by examining thousands of miRNA-target interactions from high-throughput sequencing experiments, predicted all of the targets in miRDB [16]. MiRmap (https://mirmap.ezlab.org/app/) examines feature correlations and compares their prediction potential by utilizing high-throughput experimental data from immunopurification, transcriptomics, proteomics, and polyribosome isolation experiments [17]. These predicted miRNAs were considered as EPHX3 candidate miRNAs.

### 2.5. Exploration of the starBase Database

The starBase (https://starbase.sysu.edu.cn/) provides information on the interaction between miRNAs and various RNA molecules [18]. Next, we used starBase to evaluate the correlation of miRNA-EPHX3 expression in HNSCC and the expression levels of miRNA in HNSCC patients and healthy individuals.

### 2.6. Survival Analysis Using Kaplan-Meier Plotter

The Kaplan-Meier plotter (https://kmplot.com/analysis/) can evaluate the association of 54k genes (mRNA, miRNA, and proteins) and prognosis of 21 cancer types. The tool uses meta-analysis to identify and validate survival biomarkers [19] and can be used for miRNA survival analysis. The back-end database needs manual maintenance. GEO, EGA, and TCGA provided gene expression data as well as relapse-free and overall survival information. To determine the prognostic value of genes, patient samples were divided into two groups based on different quartiles of gene expression. For the purpose of study the relationship between the expression level of miR-4713-3p and the prognosis of patients with HNSCC, we conducted a KM plotter to analyze. Log-rank *p* < 0.05 was considered statistically significant.

### 2.7. Immunomodulation Analysis

TIMER (http://timer.cistrome.org/) [20] is a web tool that facilitates analysis of tumor-infiltrating immune cells in great detail. The xcell algorithm was used to investigate the relationship between EPHX3 expression and immune cell infiltration or immune checkpoint expression in HNSC on TIMER. *p* < 0.05 indicated statistical significance.

### 2.8. Cells and Main Reagents

Nasopharyngeal carcinoma cell lines (CNE1 and CEN2) and nasopharyngeal epithelial cells (NP69) were kept at the Scientific Laboratory Centre of Hainan Medical University. 10% Gibco fetal bovine serum purchased from Life Technologies. Primary antibodies against EPHX3 and *β*-actin were acquired from Shanghai Absin Co., and the secondary antibodies labeled with horseradish peroxidase were purchased from Shanghai Biyuntian Co.

### 2.9. Western Blot to Detect the Expression Level of EPHX3 in Each Group of Cells

The cells were digested with trypsin, centrifuged, and diluted into cell suspension, inoculated into 6-well plates at 1 × 10^5^ cells/well, incubated in 5% CO2 of 37°C for 24 h. Then, the protein lysate was added; the total protein was extracted, denatured in boiling water, separated by electrophoresis, transferred, washed, and incubated for 30 min in blocking solution; and the primary antibody (1 : 500) was added and incubated overnight. Later, the secondary antibody (1 : 2000) was added, and the bands were visualized with GAPDH as internal reference. GAPDH was used as the internal reference, and the grey scale values of the bands were statistically analyzed by the ImageJ image analysis software.

### 2.10. Statistical Analysis

SPSS 19.0 and GraphPad 5.01 were used for data statistics. The *t*-test and one-way ANOVA were used for comparison between two groups and between multiple groups, respectively; *p* < 0.05 was considered statistically significant.

## 3. Results

### 3.1. EPHX3 Expression in Cancers across the Board

**Figure 1:**
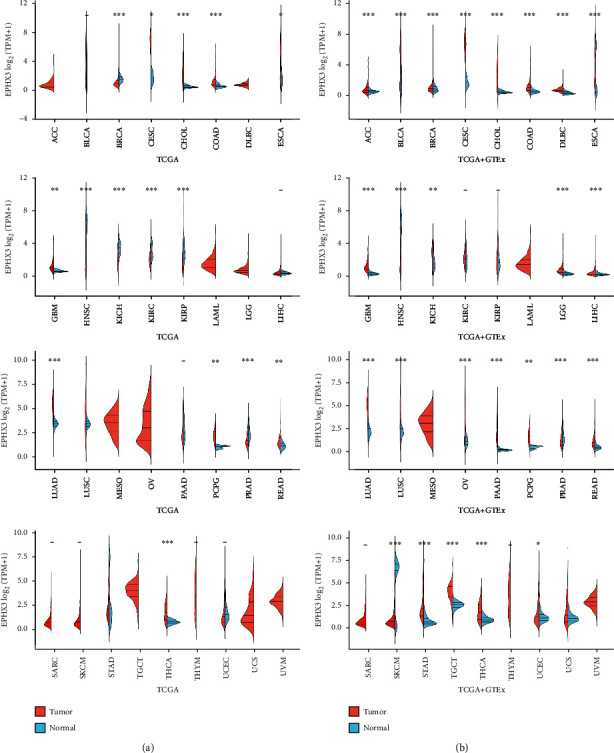
EPHX3 expression in various cancers was studied. (a) EPHX3 expression was examined in 33 human cancer types using TCGA datasets from cancer tissues and normal tissues. (b) EPHX3 expression in 33 human cancer types was compared to TCGA and GTEx normal tissues. ^∗^, ^∗∗^, and ^∗∗∗^ indicate *p* < 0.05, *p* < 0.01, and *p* < 0.001, respectively.

To understand the regulatory role of EPHX3 in cancer, we first examined its expression in 33 human cancer types on TCGA and found that EPHX3 is highly expressed in CESC, CHOL, COAD, GBM, LUAD, PCPG, READ, and THCA but poorly expressed in HNSC, BRCA, ESCA, KICH, KIRC, KIRP, and PRAD (Figure 1(a)). Next, analysis of EPHX3 expression in 33 cancers along with GETX data found that BLCA, CESC, CHOL, COAD, DLBC, GBM, LGG, LUAD, LUSC, OV, PAAD, PCPG, READ, STAD, TGCT, and THCA had high EPHX3 expression levels, while ACC, BRCA, ESCA, HNSC, KICH, LICH, PRAD, SKCM, and UCEC had low EPHX3 expression (Figure 1(b)). Studies have shown that patients with low EPHX3 expression in prostate cancer are more likely to relapse in their prognosis [8]. In Oral Squamous Cell Carcinoma, EPHX3 is closely related to lymph node involvement and secondary tumor events [21]. In addition, the downregulation of EPHX3 is related to the occurrence and development of adenoid cystic carcinoma of salivary gland [22]. These data indicate that EPHX3 is a critical regulator of tumorigenesis in the 13 cancers analyzed.

### 3.2. EPHX3's Prognosis Values in Oncogenesis

**Figure 2:**
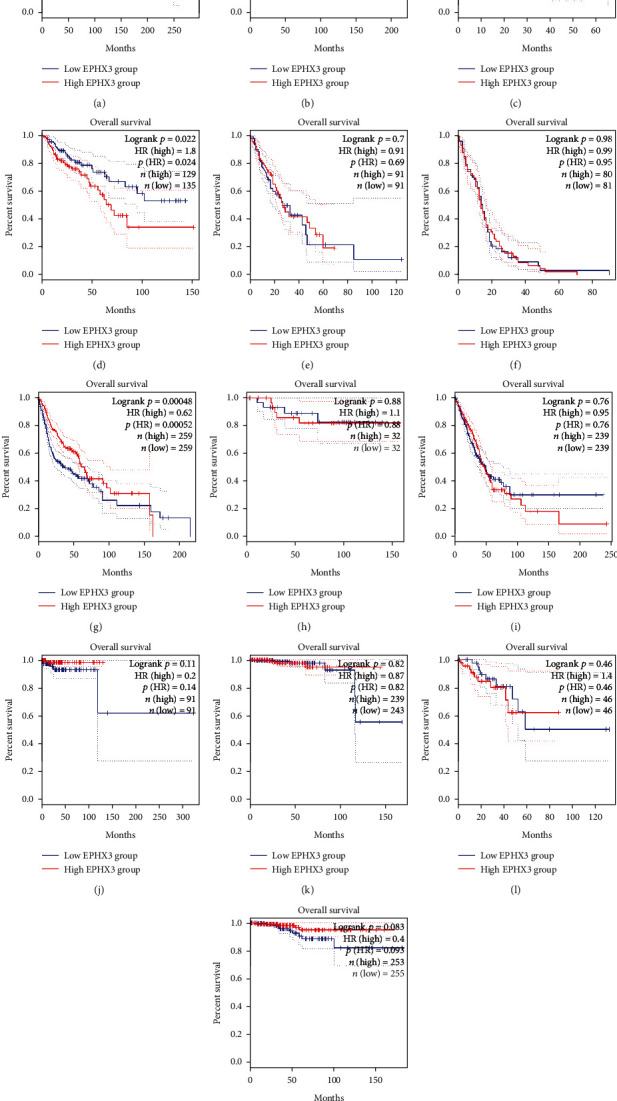
GEPIA analysis of correlation between EPHX3 and OS in various human cancers. Correlation between EPHX3 and OS in BRCA (a), CESC (b), CHOL (c), COAD (d), ESCA (e), GBM (f), HNSC (g), KICH (h), LUAD (i), PCPG (j), PRAD (k), READ (l), and THCA (m).

**Figure 3:**
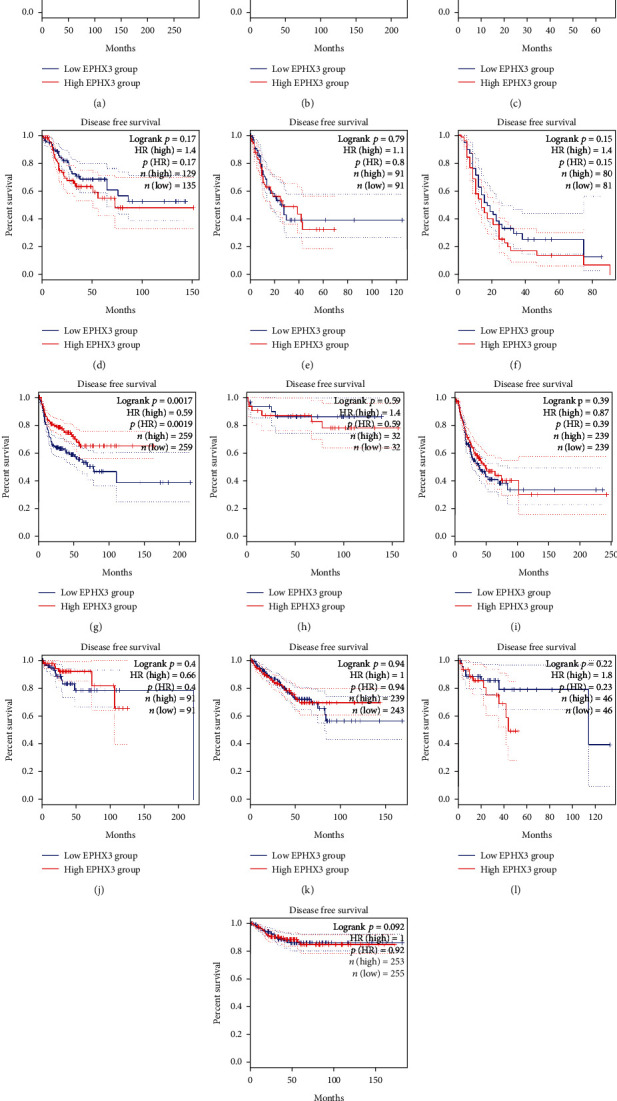
GEPIA analysis of association between EPHX3 expression and RFS in human cancers. Correlation between EPHX3 and RFS in BRCA (a), CESC (b), CHOL (c), COAD (d), ESCA (e), GBM (f), HNSC (g), KICH (h), LUAD (i), PCPG (j), PRAD (k), READ (l), and THCA (m).

Next, we examined the association between EPHX3 expression and survival in the 13 cancers. This analysis revealed that high EPHX3 levels correlate with poor overall survival (OS) in COAD patients, while in HNSC patients high EPHX3 levels correlate with favorable prognosis (Figure 2). However, high EPHX3 expression only correlated significantly with better disease free survival (DFS) in HNSCC patients but not in other cancer types (Figure 3). Thus, EPHX3 can predict adverse outcomes for HNSCC based on OS and DFS.

### 3.3. EPHX3 Functional Enrichment Analysis

**Figure 4:**
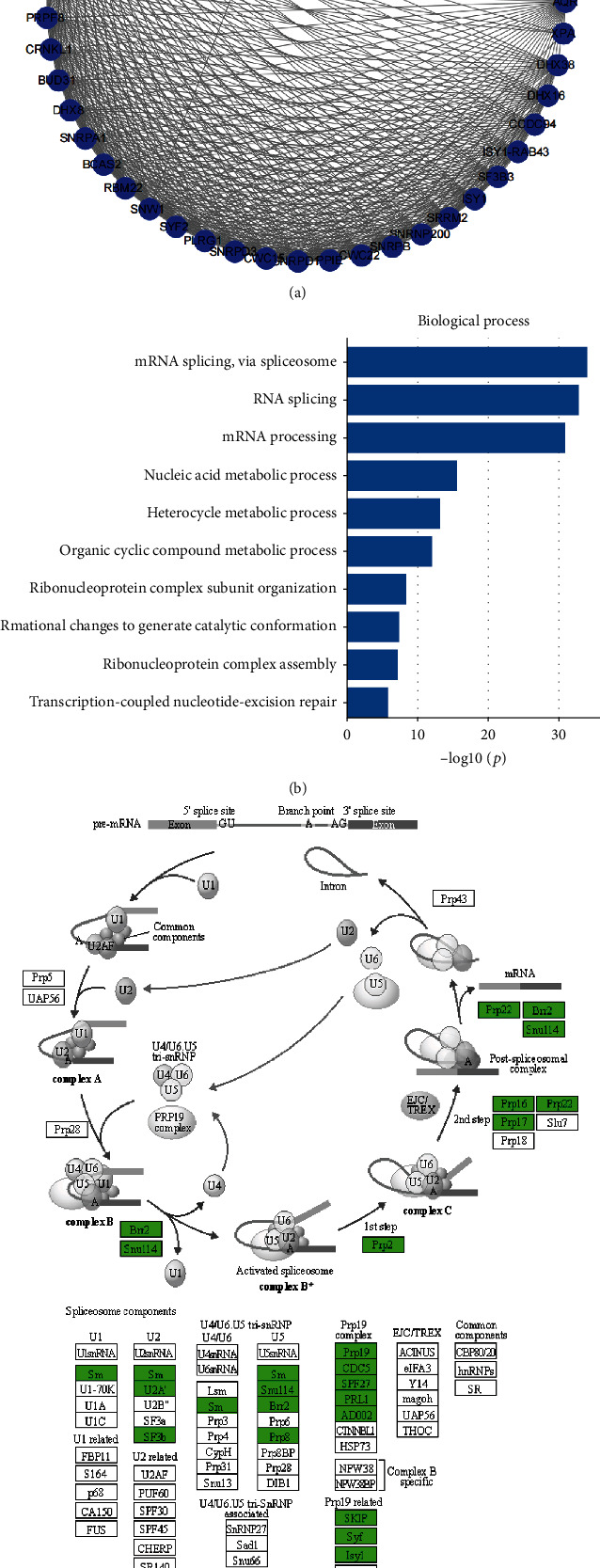
Functional analysis of genes. (a) Visualization of a PPI network of 50 related genes on Cytoscape. (b) TOP 10 GO terms associated with BP (*p* < 0.05). (c) Spliceosome enrichment pathway map (green genes indicate genes that are enriched in this pathway).

To better understand the molecular function of EPHX3, the top 50 genes closely related to EPHX3 were identified on STRING database. We also performed protein-protein interaction network construction (PPI) (Figure 4(a)), gene ontology (GO) (Figure 4(b)), and Kyoto Encyclopedia of Genes and Genomes (KEGG) (Figure 4(c)) enrichment analysis. This analysis showed that EPHX3 has a regulatory role in expression mainly through the spliceosome of mRNA.

### 3.4. Prediction of Anti-EPHX3 miRNAs

**Figure 5:**
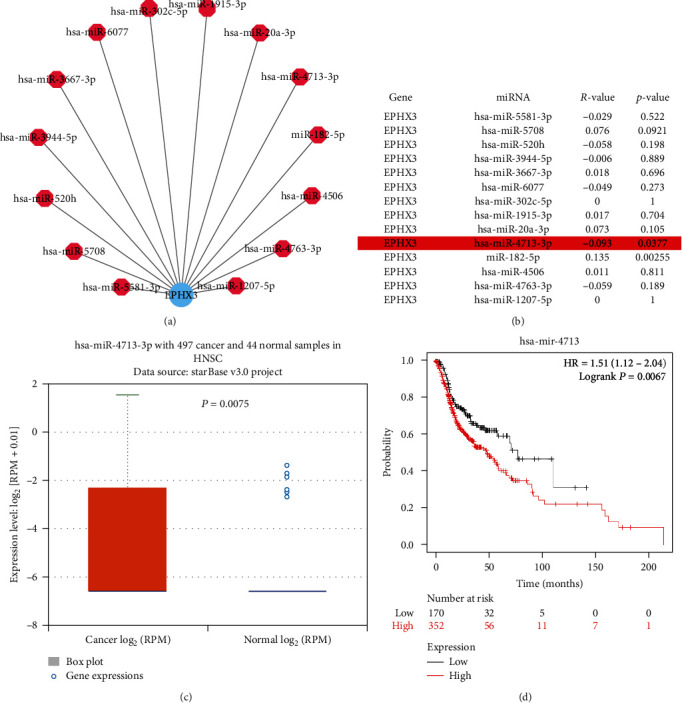
The role of miR-4713-3p in the regulation of EPHX3 in HNSC. (a) Cytoscape was used to create a miRNA-EPHX3 regulatory network. (b) starBase was used to examine correlation between the expression of predicted miRNAs and EPHX3 in HNSCC. (c) starBase analysis showed that miR-4713-3p is differentially expressed in HNSCC samples relative to normal tissues (*p* < 0.05). (d) Kaplan-Meier plotter analysis of the prognostic value of miR-4713-3p in HNSC.

An analysis of whether EPHX3 is controlled by miRNA identified 14 miRNAs that might target EPHX3 (Figure 5(a)). miRNA expression generally correlates negatively with target gene expression. Our analysis showed that in HNSCC, EPHX3 significantly and inversely correlates with miR-4713-3p expression (Figure 5(b)). Analysis of the expression of miR-4713-3p HNSCC, as well as its prognostic value, revealed that it is significantly elevated in HNSCC, and that its upregulation was linked to patient poor prognosis (Figures 5(c) and 5(d)). Together, these data highlight miR-4713-3p as the most plausible regulator of EPHX3 in HNSC.

### 3.5. Immune Cell Infiltration Analysis of EPHX3 and HNSC

**Figure 6:**
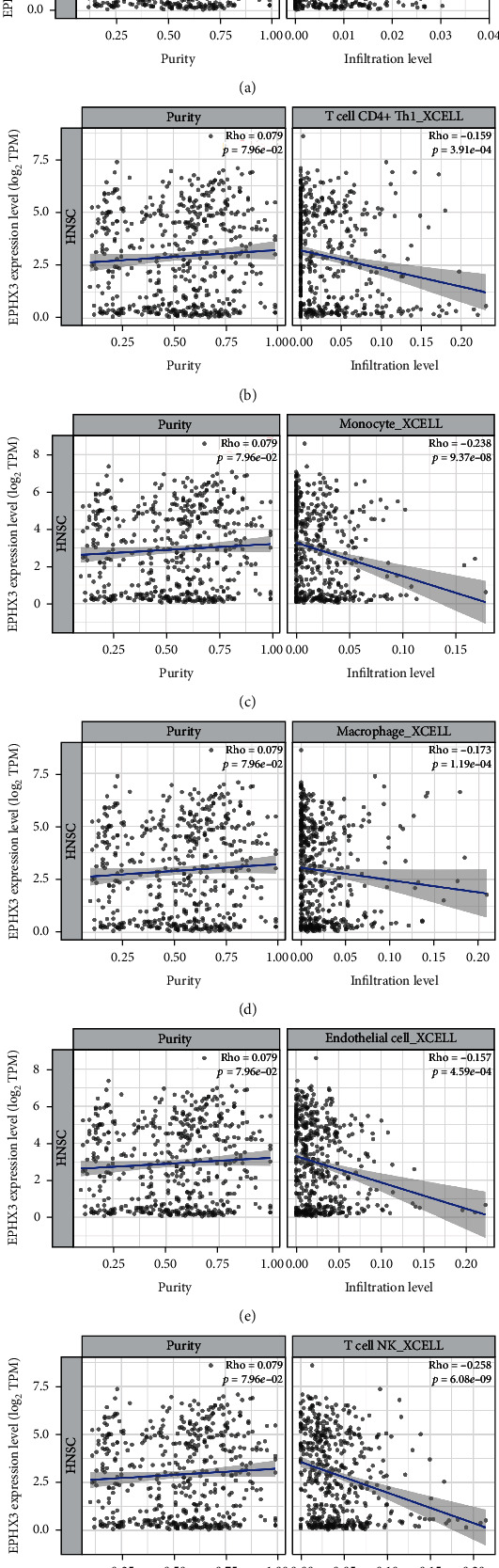
Purity adjustment for immune infiltration analysis: (a) naïve CD8+ T cells; (b) TH1 CD4+ T cells; (c) monocyte; (d) macrophage; (e) endothelial cells; (f) NK T cells.

The relationship between EPHX3 and immune cell infiltration was investigated because immune cell plays an essential regulatory function in tumorigenesis. This analysis showed that EPHX3 expression negatively correlates with infiltration by naïve CD8+ T-cells, TH1 CD4+ T-cells, monocyte, macrophages, endothelial cells, and NK T-cells (Figures 6(a)–6(f)).

### 3.6. EPHX3 and the Expression of HNSC Immune Cell Indicators Are Linked

To further investigate the role of EPHX3 in tumor immunity, we used GEPIA to determine if there is a link between EPHX3 and the expression of immune cell biomarkers in HNSCC. This analysis revealed that EPHX3 expression negatively correlated with T-cell CD8+ naïve (NCAM1), T-cell CD4+ TH1 (CD25), monocytes (CD14), macrophages (CD163, VSIG4, MS4A4A), endothelial cells (CDH5), and T-cell NK (CD56, FCGR3A) in HNSCC (Table 1).

### 3.7. Correlation Analysis of EPHX3 and HNSCC Immune Checkpoints

**Figure 7:**
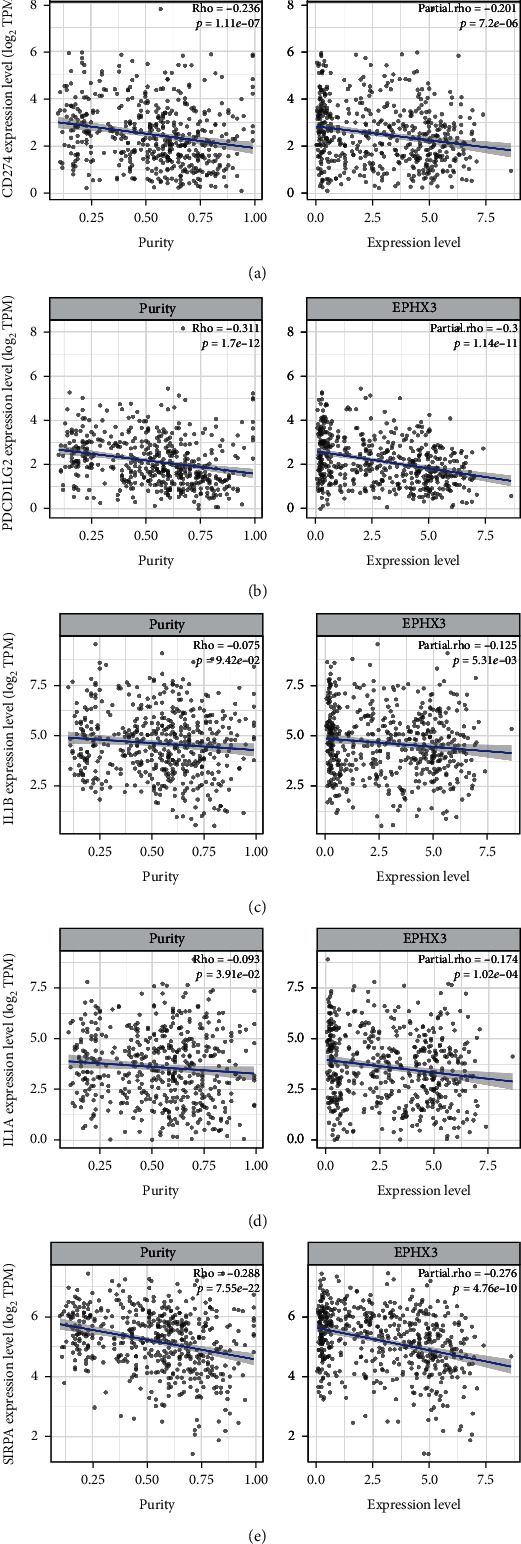
Correlation of EPHX3 expression and immune checkpoints. (a) TIMER was used to modify the Spearman correlation between EPHX3 and CD274 expression in HNSCC. (b) TIMER was used to modify the Spearman correlation between EPHX3 and PDCD1LG2 expression in HNSCC. (c) TIMER was used to modify the Spearman correlation between EPHX3 and IL1B expression in HNSCC. (d) TIMER was used to modify the Spearman correlation between EPHX3 and IL1A expression in HNSCC. (e) TIMER was used to modify the Spearman correlation between EPHX3 and SIRPA expression in HNSCC.

Important immunological checkpoints involved in tumor immune escape include CD274, IL1B, IL1A, PDCD1, PDCD1LG2, and SIRPA. These immune checkpoints are closely associated with HNSCC tumorigenesis (Figure 7(a)). Next, considering the potential tumor suppressor role of EPHX3 in HNSCC, we examined the association between EPHX3 and CD274, IL1B, IL1A, PDCD1, and PDCD1LG2. This analysis uncovered significant negative correlation between EPHX3 and these checkpoints in HNCC (Figures 7(a)–7(e)), indicating that in HNSCC, EPHX3 may play an important role in the suppression of immune escape via these checkpoints.

### 3.8. Western Blot for EPHX3 Expression

**Figure 8:**
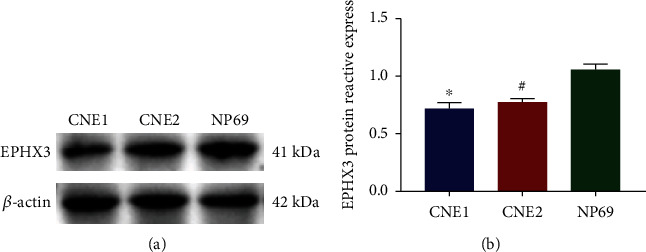
Expression of EPHX3 in nasopharyngeal carcinoma cells. (a) EPHX3 expression levels in different groups. (b) EPHX3 protein reactive expression. ^∗^*p* < 0.05 CNE1 vs. NP69, #*p* < 0.05 CNE2 vs. NP69.

The results of Western blotting experiments of each group showed that EPHX3 expression level in NPC cells in the CEN1 and CEN2 groups was lower than that in the NP69 group (Figure 8), suggesting that EPHX3 may play an important role in NPC.

## 4. Discussion

Despite current advances in the diagnosis and treatment of HNSCC, typically patients are already advanced at diagnosis, and the 5-year overall survival rate is less than 50% [23]. The reasons for HNSCC mortality include local recurrence, cervical lymphatic metastasis, and treatment failure due to resistance to standard chemotherapy [24]. Thus, effective therapeutic targets or prognostic indicators for HNSCC are urgently needed. EPHX3 is expressed and regulated in various cancers. However, its role in HNSCC is poorly understood.

To determine EPHX3 expression in various cancers, we carried out a pan-cancer analysis of EPHX3 expression on TCGA and GETX datasets. In data analysis and cell experiments, we have confirmed the low expression of EPHX3 in nasopharyngeal carcinoma cells, suggesting that EPHX3 may play an important role in nasopharyngeal carcinoma. Next, analysis of OS and RFS data revealed that EPHX3 can be used as a prognosis marker for HNSCC and that HNSCC patients with low levels of EPHX3 had worse prognosis. Studies on gastric [6] and prostate [25] cancer have shown that decreased EPHX3 expression promotes cancer recurrence while lowering patient survival.

To understand the molecular functional of EPHX3 better, we used STRING analysis to identify the top 50 genes closely associated with EPHX3 and subjected them to GO (BP) and KEGG enrichment analyses. This analyses showed that EPHX3 is mainly involved in the spliceosome pathway to regulate the expression of other genes. The spliceosome is made of five distinct ribonucleoprotein (RNP) subunits and numerous protein cofactors [26, 27]. Impaired spliceosome function is implicated in cancer development mainly due to altered splicing of regulatory sequences on oncogenes [28, 29] and alterations in the expression of mutant genes [30–32]. Splicing requires multiple proteins and RNA interactions and is directed by many trans-acting proteins that are themselves regulated by posttranslational modifications and protein/RNA interactions. This allows manipulation of the spliceosome for therapeutic purposes [33]. However, the spliceosome processes need to be further explored.

Numerous cellular processes, including development, differentiation, proliferation, transcription, posttranscriptional modification, apoptosis, and metabolism, are modulated by ncRNAs [17, 34, 35] and long ncRNA (lncRNA). Dysregulation of ncRNA is implicated in various diseases, including cancers [36–38]. A search for miRNAs that bind to EPHX3 on TargetScan, TarBase, MIRDB, and miRmap uncovered 14 miRNAs. Correlation, expression, and survival analyses identified miRNA-4713 as the most plausible miRNA against EPHX3 in HNSC.

Many studies show that immune infiltration affects the efficacy of chemoradiotherapy and immunotherapy, as well as cancer prognosis [39–41]. Our data show that in HNSC, EPHX3 negatively correlates with infiltration by various immune cells, including naive CD8+ T cells, TH1 CD4+ T cells, monocytes, macrophages, endothelial cells, and NK T cells. Additionally, EPHX3 had significant negative correlation with these infiltrating immune cells. These findings indicate that EPHX3 might have anticancer effects in HNSCC through regulation of immune cell infiltration.

Successful immunotherapy depends not only on the presence of a sufficient level of immune cell infiltration of the tumor microenvironment but also on the expression of immunological checkpoints [42]. Our analysis of the relationship between EPHX3 and immune checkpoints revealed that low level of EPHX3 was closely associated with CD274, IL1B, IL1A, PDCD1, PDCD1LG2, and SIRPA in HNSCC, indicating that targeting EPHX3 may improve the efficacy of immunotherapy in HNSC.

## 5. Conclusions

**Figure 9:**
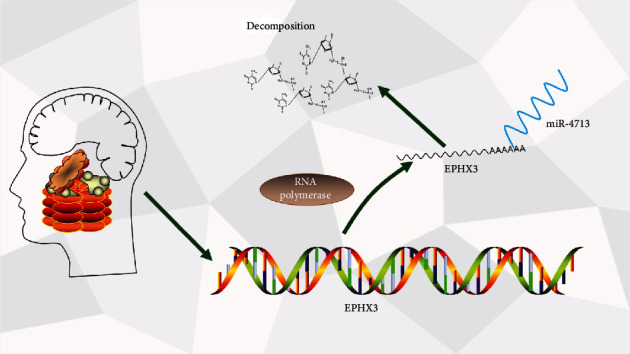
The model of miR-4713-EPHX3 axis in carcinogenesis of HNSCC.

In conclusion, our analyses indicate that poor EPHX3 expression in HNSC contributes to poor patient prognosis. Furthermore, we find that EPHX3 regulates gene expression mainly through spliceosome function. We show that in HNSC, EPHX3 is regulated by miR-4713 (Figure 9). Our data also show that EPHX3 may exert its anticancer effects by reducing tumor immune cell infiltration and immune checkpoint expression. However, these results need further investigation.

## Figures and Tables

**Table 1:** Analysis of correlation between EPHX3 and HNSCC immune cell biomarkers (^∗^ indicates *p* < 0.05).

Immune cell	Biomarker	*R* value	*p* value
CD8+ T cell	CD8A	0.025	0.56
	NCAM1	-0.11	0.014^∗^
CD4 + T cell	CD4	-0.013	0.76
	CD25	-0.092	0.037^∗^
Manphage	NOS2	0.27	0.27
	CD163	-0.14	0.002^∗^
	VSIG4	-0.11	0.12
	MS4A4A	-0.11	0.01^∗^
NK T cell	CD56	-0.11	0.014^∗^
	FCGR3A	-0.14	0.0011^∗^
Endothelial cell	CD34	−0.06	0.17
	CDH5	−0.095	0.031^∗^
Monocyte	CD14	-0.14	0.0015^∗^

## Data Availability

All data generated and analyzed during this study are included in this published article and are available on request.

## Abbreviations

ACC: Adrenocortical carcinoma
BLCA: Bladder urothelial carcinoma
BRCA: Breast invasive carcinoma
CESC: Cervical squamous cell carcinoma and endocervical adenocarcinoma
CHOL: Cholangiocarcinoma
OAD: Colon adenocarcinoma
DLBC: Lymphoid neoplasm diffuse large B-cell lymphoma
ESCA: Esophageal carcinoma
GBM: Glioblastoma multiforme
HNSC: Head and neck squamous cell carcinoma
KICH: Kidney chromophobe
KIRC: Kidney renal clear cell carcinoma
KIRP: Kidney renal papillary cell carcinoma
LAML: Acute myeloid leukemia
LGG: Brain lower grade glioma
LIHC: Liver hepatocellular carcinoma
LUAD: Lung adenocarcinoma
LUSC: Lung squamous cell carcinoma
MESO: Mesothelioma
OV: Ovarian serous cystadenocarcinoma
PAAD: Pancreatic adenocarcinoma
PCPG: Pheochromocytoma and paraganglioma
PRAD: Prostate adenocarcinoma
READ: Rectum adenocarcinoma
SARC: Sarcoma
SKCM: Skin cutaneous melanoma
STAD: Stomach adenocarcinoma
STES: Stomach and esophageal carcinoma
TGCT: Testicular germ cell tumors
THCA: Thyroid carcinoma
THYM: Thymoma
UCEC: Uterine corpus endometrial carcinoma
UCS: Uterine carcinosarcoma
UVM: Uveal melanoma.

## References

[1] Sung H., Ferlay J., Siegel R. L. (2021). Global cancer statistics 2020: GLOBOCAN estimates of incidence and mortality worldwide for 36 cancers in 185 countries. *CA: a Cancer Journal for Clinicians*.

[2] Johnson D. E., Burtness B., Leemans C. R., Lui V. W. Y., Bauman J. E., Grandis J. R. (2020). Head and neck squamous cell carcinoma. *Nature Reviews Disease Primers*.

[3] Elkashty O. A., Abu Elghanam G., Su X., Liu Y., Chauvin P. J., Tran S. D. (2020). Cancer stem cells enrichment with surface markers CD271 and CD44 in human head and neck squamous cell carcinomas. *Carcinogenesis*.

[4] von Witzleben A., Wang C., Laban S., Savelyeva N., Ottensmeier C. H. (2020). HNSCC: tumour antigens and their targeting by immunotherapy. *Cells*.

[5] Decker M., Adamska M., Cronin A. (2012). EH3 (ABHD9): the first member of a new epoxide hydrolase family with high activity for fatty acid epoxides. *Journal of Lipid Research*.

[6] Yamashita S., Tsujino Y., Moriguchi K., Tatematsu M., Ushijima T. (2006). Chemical genomic screening for methylation-silenced genes in gastric cancer cell lines using 5-aza-2′-deoxycytidine treatment and oligonucleotide microarray. *Cancer Science*.

[7] Furuta J., Nobeyama Y., Umebayashi Y., Otsuka F., Kikuchi K., Ushijima T. (2006). Silencing of Peroxiredoxin 2 and aberrant methylation of 33 CpG islands in putative promoter regions in human malignant melanomas. *Cancer Research*.

[8] Cottrell S., Jung K., Kristiansen G. (2007). Discovery and validation of 3 novel DNA methylation markers of prostate cancer prognosis. *The Journal of Urology*.

[9] Panigrahy D., Edin M. L., Lee C. R. (2012). Epoxyeicosanoids stimulate multiorgan metastasis and tumor dormancy escape in mice. *The Journal of Clinical Investigation*.

[10] Fleming I. (2007). Epoxyeicosatrienoic acids, cell signaling and angiogenesis. *Prostaglandins & Other Lipid Mediators*.

[11] Frost F. G., Cherukuri P. F., Milanovich S., Boerkoel C. F. (2020). Pan-cancer RNA-seq data stratifies tumours by some hallmarks of cancer. *Journal of Cellular and Molecular Medicine*.

[12] Szklarczyk D., Morris J. H., Cook H. (2017). The STRING database in 2017: quality-controlled protein-protein association networks, made broadly accessible. *Nucleic Acids Research*.

[13] Tang Z., Li C., Kang B., Gao G., Li C., Zhang Z. (2017). GEPIA: a web server for cancer and normal gene expression profiling and interactive analyses. *Nucleic Acids Research*.

[14] Lewis B. P., Burge C. B., Bartel D. P. (2005). Conserved seed pairing, often flanked by adenosines, indicates that thousands of human genes are microRNA targets. *Cell*.

[15] Karagkouni D., Paraskevopoulou M. D., Chatzopoulos S. (2018). DIANA-TarBase v8: a decade-long collection of experimentally supported miRNA-gene interactions. *Nucleic Acids Research*.

[16] Chen Y., Wang X. (2020). miRDB: an online database for prediction of functional microRNA targets. *Nucleic Acids Research*.

[17] Adams B. D., Parsons C., Walker L., Zhang W. C., Slack F. J. (2017). Targeting noncoding RNAs in disease. *The Journal of Clinical Investigation*.

[18] Li J. H., Liu S., Zhou H., Qu L. H., Yang J. H. (2014). starBase v2.0: decoding miRNA-ceRNA, miRNA-ncRNA and protein-RNA interaction networks from large-scale CLIP-Seq data. *Nucleic Acids Research*.

[19] Pan J. H., Zhou H., Cooper L. (2019). LAYN is a prognostic biomarker and correlated with immune infiltrates in gastric and colon cancers. *Frontiers in Immunology*.

[20] Li T., Fan J., Wang B. (2017). TIMER: a web server for comprehensive analysis of tumor-infiltrating immune cells. *Cancer Research*.

[21] Gissi D. B., Fabbri V. P., Gabusi A. (2020). Pre-operative evaluation of DNA methylation profile in oral squamous cell carcinoma can predict tumor aggressive potential. *International Journal of Molecular Sciences*.

[22] Bell A., Bell D., Weber R. S., El-Naggar A. K. (2011). CpG island methylation profiling in human salivary gland adenoid cystic carcinoma. *Cancer*.

[23] González-Arriagada W. A., Lozano-Burgos C., Zúñiga-Moreta R., González-Díaz P., Coletta R. D. (2018). Clinicopathological significance of chemokine receptor (CCR1, CCR3, CCR4, CCR5, CCR7 and CXCR4) expression in head and neck squamous cell carcinomas. *Journal of Oral Pathology & Medicine*.

[24] Shield K. D., Ferlay J., Jemal A. (2017). The global incidence of lip, oral cavity, and pharyngeal cancers by subsite in 2012. *CA: a Cancer Journal for Clinicians*.

[25] Stott-Miller M., Zhao S., Wright J. L. (2014). Validation study of genes with hypermethylated promoter regions associated with prostate cancer recurrence. *Cancer Epidemiology and Prevention Biomarkers*.

[26] Will C. L., Lührmann R. (2011). Spliceosome structure and function. *Cold Spring Harbor Perspectives in Biology*.

[27] Jurica M. S., Moore M. J. (2003). Pre-mRNA splicing: awash in a sea of proteins. *Molecular Cell*.

[28] Supek F., Miñana B., Valcárcel J., Gabaldón T., Lehner B. (2014). Synonymous mutations frequently act as driver mutations in human cancers. *Cell*.

[29] Jung H., Lee D., Lee J. (2015). Intron retention is a widespread mechanism of tumor-suppressor inactivation. *Nature Genetics*.

[30] Karni R., de Stanchina E., Lowe S. W., Sinha R., Mu D., Krainer A. R. (2007). The gene encoding the splicing factor SF2/ASF is a proto-oncogene. *Nature Structural & Molecular Biology*.

[31] Jensen M. A., Wilkinson J. E., Krainer A. R. (2014). Splicing factor SRSF6 promotes hyperplasia of sensitized skin. *Nature Structural & Molecular Biology*.

[32] Anczuków O., Rosenberg A. Z., Akerman M. (2012). The splicing factor SRSF1 regulates apoptosis and proliferation to promote mammary epithelial cell transformation. *Nature Structural & Molecular Biology*.

[33] Lee S. C., Abdel-Wahab O. (2016). Therapeutic targeting of splicing in cancer. *Nature Medicine*.

[34] Zhu S., Wang J., He Y., Meng N., Yan G. R. (2018). Peptides/proteins encoded by non-coding RNA: a novel resource bank for drug targets and biomarkers. *Frontiers in Pharmacology*.

[35] Wang J., Zhu S., Meng N., He Y., Lu R., Yan G. R. (2019). ncRNA-encoded peptides or proteins and cancer. *Molecular therapy : the journal of the American Society of Gene Therapy*.

[36] Jiang H., Huang G., Zhao N. (2018). Long non-coding RNA TPT1-AS1 promotes cell growth and metastasis in cervical cancer via acting AS a sponge for miR-324-5p. *Journal of experimental & clinical cancer research*.

[37] Xiong H., Chen R., Liu S., Lin Q., Chen H., Jiang Q. (2018). Retracted: MicroRNA-183 induces epithelial-mesenchymal transition and promotes endometrial cancer cell migration and invasion in by targeting CPEB1. *Journal of Cellular Biochemistry*.

[38] Huang J. Z., Chen M., Zeng M. (2016). Down-regulation of TRPS1 stimulates epithelial–mesenchymal transition and metastasis through repression of FOXA1. *The Journal of Pathology*.

[39] Waniczek D., Lorenc Z., Śnietura M., Wesecki M., Kopec A., Muc-Wierzgoń M. (2017). Tumor-associated macrophages and regulatory T cells infiltration and the clinical outcome in colorectal cancer. *Archivum Immunologiae et Therapiae Experimentalis*.

[40] Zhang H., Liu H., Shen Z. (2018). Tumor-infiltrating neutrophils is prognostic and predictive for postoperative adjuvant chemotherapy benefit in patients with gastric cancer. *Annals of Surgery*.

[41] Lyu L., Yao J., Wang M. (2020). Overexpressed pseudogene HLA-DPB2 promotes tumor immune infiltrates by regulating HLA-DPB1 and indicates a better prognosis in breast cancer. *Frontiers in Oncology*.

[42] Chae Y. K., Arya A., Iams W. (2018). Current landscape and future of dual anti-CTLA4 and PD-1/PD-L1 blockade immunotherapy in cancer; lessons learned from clinical trials with melanoma and non-small cell lung cancer (NSCLC). *Journal for Immunotherapy of Cancer*.

